# Ossifying Fibroma of Non-odontogenic Origin: A Fibro-osseous Lesion in the Craniofacial Skeleton to be (Re-)considered

**DOI:** 10.1007/s12105-021-01351-3

**Published:** 2021-06-26

**Authors:** Daniel Baumhoer, Simon Haefliger, Baptiste Ameline, Wolfgang Hartmann, Fernanda Amary, Arjen Cleven, Michael J. Klein, Lester D. R. Thompson, Dorothee Harder, Paul O’Donnell

**Affiliations:** 1 Bone Tumor Reference Centre, Institute of Medical Genetics and Pathology, University Hospital Basel, University of Basel, Schoenbeinstrasse 40, 4031 Basel, Switzerland; 2 Division of Translational Pathology, Gerhard-Domagk-Institut of Pathology, University Hospital Münster, Albert-Schweitzer-Campus 1, 48149 Münster, Germany; 3 Cellular and Molecular Pathology, Royal National Orthopaedic Hospital, Brockley Hill, Stanmore, Greater London, HA7 4LP UK; 4 Department of Pathology, Leiden University Medical Center, Leiden, The Netherlands; 5 Hospital for Special Surgery, 535 E 86th St, New York, NY 10021 USA; 6 Department of Pathology, Woodland Hills Medical Center, Woodland Hills, CA USA; 7 Department of Radiology, University Hospital Basel, University of Basel, Petersgraben 4, 4031 Basel, Switzerland; 8 Department of Radiology, Royal National Orthopaedic Hospital, Brockley Hill, Stanmore, Greater London, HA7 4LP UK; 9 Cancer Institute, University College London, Huntley Street, London, WC1E 6BT UK

**Keywords:** Fibro-osseous lesion, Cemento-osseous dysplasia, Cemento-ossifying fibroma, Fibrous dysplasia, Central low-grade osteosarcoma, Craniofacial

## Abstract

In the cranio-facial skeleton, a heterogeneous group of well characterized fibro-osseous lesions can be distinguished. Whereas fibrous dysplasia can affect any skeletal bone, ossifying fibroma and cemento-osseous dysplasia exclusively develop in the cranio-facial region, with most subtypes restricted to the tooth bearing areas of the jaws. Herein we present a series of 20 fibro-osseous lesions that developed mostly in the frontal bone and in the mandible, presenting as expansile intramedullary tumors with a unique histologic appearance and an indolent clinical course. We provide evidence that these tumors are distinct from the categories included in the WHO classification and are therefore currently unclassifiable. The definition of cemento-ossifying fibroma as an odontogenic neoplasm developing only in close proximity to teeth should be re-considered and incorporate also extragnathic lesions as shown here.

**Supplementary Information:**

The online version contains supplementary material available at 10.1007/s12105-021-01351-3.

## Introduction

Fibro-osseous lesions of the craniofacial skeleton comprise a distinct group of benign tumors that differ in clinical presentation, imaging, and morphology. Traditionally, craniofacial fibrous dysplasia (CFD), ossifying fibroma (OF), and cemento-osseous dysplasia (COD) are distinguished [5–7]. Whereas fibrous dysplasia (FD) can occur anywhere in the skeleton, OF and COD are exclusively found in the maxillofacial bones. Since all these lesions can show significant morphological overlap, particularly on small biopsy samples, the clinical context and the corresponding imaging are required for accurate classification [1, 9, 12].

FD is caused by a postzygotic missense mutation in the *GNAS* gene and can involve single (monostotic) or multiple bones (polyostotic). In the craniofacial skeleton, typically, adjacent bones can be affected and whilst this is still considered a monostotic disease, it represents a unique finding in this site (CFD). CFD typically expands the affected bone(s) and on imaging studies shows a mixed lytic-ground-glass appearance; molecular confirmation of the diagnosis can be achieved by *GNAS* mutation testing [10, 11]. OF comprises a conventional subtype (cemento-ossifying fibroma) which is considered an odontogenic neoplasm (COF) along with two juvenile subtypes (juvenile psammomatoid/trabecular ossifying fibroma, JPOF/JTOF) that can also occur at extragnathic sites [4]. Radiographically, all OF are well defined, expansile lesions with irregular matrix mineralization [1, 9]. COD typically develops in close proximity to teeth roots and can be stratified into periapical, focal, and florid subtypes depending on location and growth patterns. It is usually non-expansile, may occur multifocally and adjacent lesions can merge into larger conglomerates [4].

According to the 2017 WHO classification of head and neck tumors, COF and COD are defined as lesions of odontogenic origin and therefore are restricted to the tooth-bearing areas of the jaws [4–7]. Here, we present a series of fibro-osseous lesions with a propensity for the frontal bone that share a unique histology and followed an indolent clinical course. We compare our findings with cases that arose in the nasal bone, the maxillary sinus and the jaws which appeared histologically similar. The majority of these tumors did not arise in close proximity to teeth or developed inferior to the mandibular canal arguing against an odontogenic origin. All tumors shown are unclassifiable according to the current WHO classification. We therefore propose to revise and broaden the definition of cemento-ossifying fibroma in the 5th edition of the WHO classification and suggest to use the term conventional ossifying fibroma to summarize odontogenic and non-odontogenic tumors. Alternatively, a new and separate category of non-odontogenic ossifying fibroma should be considered.

## Material and Methods

In the consultation service of the Basel Bone Tumor Reference Center (BTRC) and DOESAK (German–Austrian–Swiss Working Group on Maxillofacial Tumors) reference registry, 20 cases of currently unclassifiable fibro-osseous lesions were assembled, correlated with clinical (follow-up) data, and corresponding imaging. In five cases, gene panel sequencing (Oncomine™ Comprehensive), Archer™ FusionPlex™ testing as well as methylation and copy number profiling, were carried out following routine protocols (for details see supplement). Four additional cases underwent *GNAS* mutation analysis using a smaller gene panel (Oncomine™ Colon Panel). The study was approved at the University Hospital Basel, following the approval of the Ethical Committee for Mutational Analysis of Anonymized Samples (“Ethikkommission beider Basel” ref. 274/12).

## Results

The patients’ average age was 44 years (range 27–74 years, median 39 years), with 14 males and 6 females. Ten cases occurred in the frontal bone with a propensity for the zygomatic process (n = 6), seven lesions developed in the mandible, and three were identified in the maxillary sinus or nasal bone (Table 1). Clinically, most cases were detected as incidental imaging findings conducted due to various reasons, with three patients reporting painless swellings. Follow-up information was available for eight patients (range 1–31 months), with no disease progression or recurrence identified.

**Table 1:** Patient and lesion characteristics

Case	Sex	Age	Site	Specimen	Greatest single dimension	Additional tests
1	Female	38	Frontal bone, ZP	Curettage	27 mm	IP
2	Female	47	Frontal bone, ZP	Curettage	39 mm	–
3	Male	48	Frontal bone, ZP	Curettage	33 mm	IP, PSeq2, Fx-, Meth + CNV
4	Male	34	Frontal bone, ZP	Curettage	35 mm	–
5	Male	34	Frontal bone, ZP	Biopsy	25 mm	–
6	Male	62	Frontal bone, ZP	Curettage	18 mm	–
7	Male	30	Frontal bone	Curettage	21 mm	IP, PSeq1-
8	Female	29	Frontal sinus	Curettage	17 mm	IP
9	Male	29	Frontal sinus	Curettage	27 mm	IP, Fx-
10	Female	28	Frontal sinus	Curettage	8 mm	IP, PSeq2, Fx-, Meth + CNV
11	Male	63	Nasal bone	NA	NA	–
12	Female	27	Maxillary sinus	Biopsy	25 mm	–
13	Male	58	Left distal mandible^a^	Curettage	6 mm	IP, PSeq1-
14	Male	44	Left distal mandible^b^	Biopsy	18 mm	IP, PSeq1-
15	Male	74	Left distal mandible^a^	Biopsy	24 mm	IP, PSeq2, Fx-, Meth + CNV
16	Male	32	Left distal mandible^b^	Biopsy	20 mm	IP, PSeq2, Fx-, Meth + CNV
17	Male	59	Right distal mandible^b^	Biopsy	12 mm	IP
18	Male	65	Right distal maxilla^a^	Curettage	10 mm	IP
19	Female	32	Angle of the right mandible^a^	Curettage	9 mm	IP, PSeq1-
20	Male	40	Right distal mandible^a^	Curettage	32 mm	IP, PSeq2, Fx-, Meth + CNV

*ZP* zygomatic process, *NA* not available, *IP* immunophenotyping, *PSeq1*- no mutations detected using Oncomine™ Colon Panel Sequencing (14 genes incl. GNAS), *PSeq2* Oncomine™ Comprehensive Panel Sequencing (135 genes incl. GNAS), *Fx*- no fusion transcripts detected using Archer^TM^FusionPlex™ Custom Panel Sequencing (53 genes and fusion partners), *Meth* + *CNV* methylation and copy number profiling

^a^Not associated with teeth

^b^Adjacent to teeth but not centered around the root

### Histological Findings

#### Frontal Lesions

**Fig. 1:**
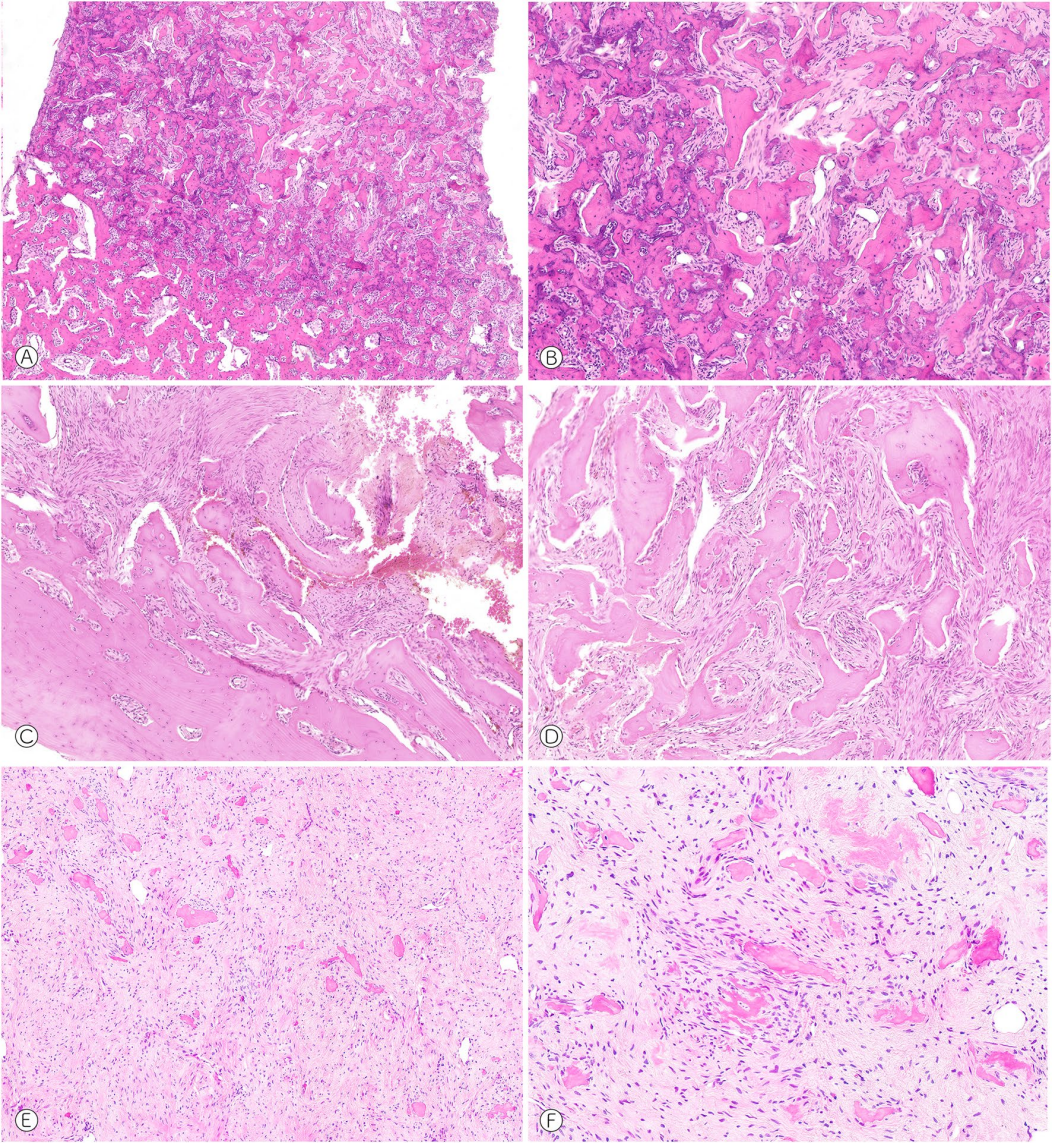
Fibro-osseous tumors of the frontal bone. **A** Fibro-osseous lesion showing a peripheral rim of reactive new bone formation (case 7, HE, × 50). **B** Higher magnification reveals dense matrix formation with incomplete lamellar maturation and a well vascularized stroma (case 7, HE, × 100). **C** The lesional matrix merges with the preexisting cortical bone (case 1, HE, × 50). **D** Slim spindle cells with moderate cellularity and more plump and trabecular woven bone (case 1, HE, × 50). **E** and **F** Rather hypocellular background of monomorphic spindle cells with few and immature new bone formation, partly lace-like (case 8, HE, × 50, × 100)

**Fig. 2:**
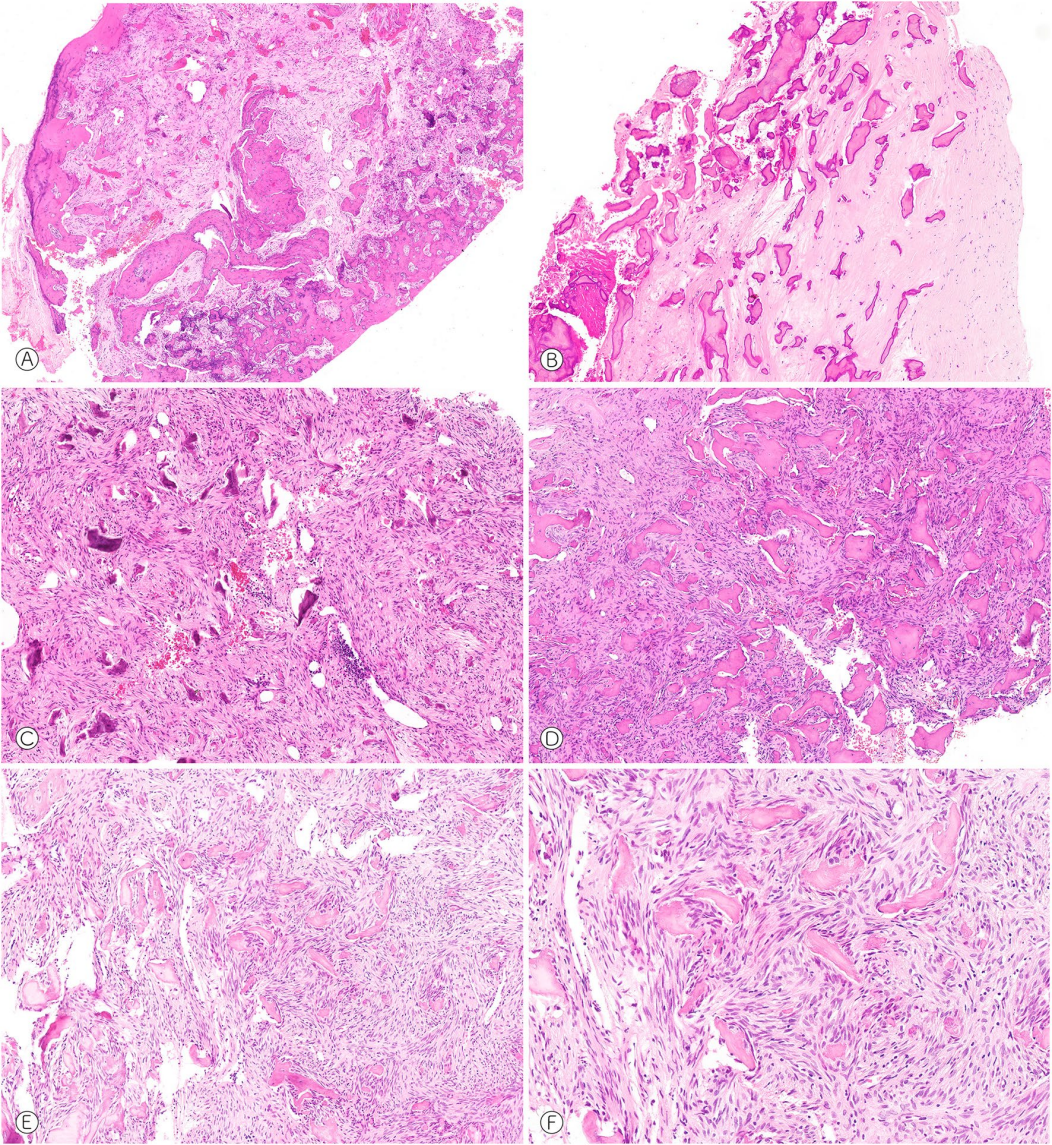
Fibro-osseous tumors of the nasal bone, maxillary sinus and jaws. **A** Plump woven bone formation and moderately cellular fibroblastic spindle cells (case 11, HE, × 50). **B** Densely collagenized and hypocellular background with partly devitalized matrix deposits potentially due to regressive changes (case 2, HE, × 50). **C** Hypercellular and slender spindle cells with immature and cementum-like matrix formation (case 13, HE, × 50). **D** Smaller fragments of hypocellular matrix deposition without osteoblastic rimming (case 20, HE, × 50). **E** and **F** Fibroblastic spindle cells encompassing immature fragments of hypocellular woven bone (case 19, HE, × 50, × 100)

All cases demonstrated a fibro-osseous morphology consisting of immature and irregular formation of bone matrix surrounded by a collagenous stroma with fibroblastic appearing spindle cells. The stromal component varied in cellular content ranging from cases with dense (Fig. 1C, D), moderate (Fig. 1A, B) and low (Fig. 2B) cellularity, the latter possibly being affected by regressive changes, similar to what can be observed in long-standing fibrous dysplasia. At higher magnification, the lesional cells revealed indistinct cell borders as well as elongated and tapered nuclei with evenly distributed chromatin. All lesions appeared monomorphic with little variation in cellular size and shape. Pleomorphism, high mitotic rate, and increased inflammation were absent. The spindle cells were arranged haphazardly and occasionally in short fascicles, whorls or even storiform patterns, sometimes resembling neural differentiation. The vascularity was sparse with only few and inconspicuous capillaries visible in most cases. The osseous component in all cases showed evenly distributed hard tissue formation that primarily consisted of immature woven bone. Whereas in some cases, complex patterns with interconnected trabeculae were perceivable, the majority of lesions showed more isolated matrix deposits. Notably, the woven bone lacked osteoblastic rimming and was particularly paucicellular, in some cases resembling the cement-like matrix of COF and COD (Figs. 1E, F, 2A). Some cases contained preexisting cortical bone that was in direct continuity and fused with the lesional matrix (Figs. 1C, 2A). One case showed a zone of reactive new bone formation at the periphery of the lesion, similar to what is commonly observed around the nidus of an osteoid osteoma (Fig. 1A). Some cases included dystrophic calcifications with secondary ossification as occasionally seen in soft tissue or fatty bone marrow necrosis (Fig. 1F). A peculiar finding in few cases were smaller fragments of lamellar appearing bone intermingled with cementum-like mineralizations and woven bone (Fig. 1B). This finding was different from the osteodestructive growth of a malignant tumor and more reminiscent of partial lamellar maturation of the lesional woven bone.

#### Lesions of the Nasal Bone, Maxillary Sinus and Jaws

The tumors in other anatomic sites showed a strikingly similar morphology (Fig. 2C–F). Again, monomorphic spindle cells arranged in a patternless pattern enclosed mineralized and immature matrix that consisted of isolated trabecular woven bone and cement-like deposits. Some cases showed tapered and particularly slender nuclei (Fig. 2C, D), cellular atypia or an increased mitotic rate were absent. Morphologically, some cases in the jaws presented with features reminiscent of COF and COD, although the globular mineralized masses (sometimes referred to as ginger root-like) typically observed in COD with advanced maturation were not seen. There was also no prominent osteoblastic rimming or a stromal verge separating preexisting bone and lesional matrix as commonly observed in COF. Markedly different to COF and COD was the male predilection (seven male vs one female patient) and the age distribution (average age 51 years, range 32–74 years) of the gnathic tumors. Furthermore, 5/8 tumors of the mandible and maxilla did not show any association to teeth and the remaining three were not centered around or in close proximity to a dental root.

### Imaging Findings

Lesions occurred in the frontal bone and mandible with similar frequency. The maxilla was uncommonly affected.

#### Frontal Lesions (Imaging Available for Eight Cases)

**Fig. 3:**
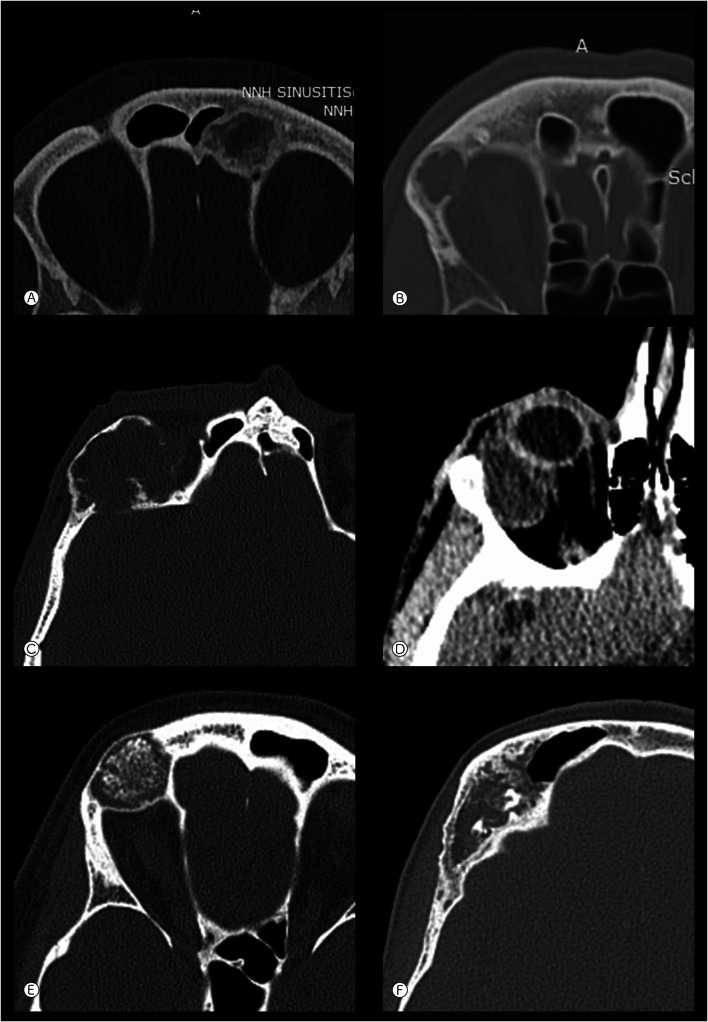
Frontal lesions. **A** Left frontal tumor expanding into the frontal sinus, showing thick peripheral sclerosis, within which is an irregular rim of increased density (case 7). **B** Right frontal tumor with mild bone expansion, a small focus of cortical destruction abutting the orbit and faint amorphous calcification (case 6). **C** and **D** Right frontal tumor with cortical destruction (**C** bone window), and, on a more inferior section, a mass in the orbit (**D** soft tissue windows) (case 4). **E** Right frontal tumor showing marked expansile remodeling and punctate mineralization (case 1). **F** Right frontal tumor containing irregular and curvilinear calcification (case 2) (all axial CT)

Most cases were intramedullary, with two cases arising within the frontal sinus. Intramedullary tumors showed variable appearances, but in general there was a well-defined lucent focus causing expansile bony remodeling. The lesions often showed a dense peripheral margin (n = 5) (Fig. 3A), sometimes with surrounding medullary sclerosis (n = 2). There was cortical thinning, occasional lobular cortical scalloping, with two cases showing cortical destruction (Fig. 3B). Expansion into sinuses or the orbit was noted in the two cases where the cortex was destroyed: there was extension of tumor into the orbit, displacing the globe in one case (Fig. 3C, D). Tumors were usually isodense but various patterns of matrix mineralization [punctate (Fig. 3E), curvilinear, and irregular calcification (Fig. 3F), faint ground glass density] were also identified. One of the sinus tumors also showed a lucent lesion with peripheral density and thinning of the surrounding bone, similar to intramedullary lesions (case 2). The other frontal sinus tumor was anterior, with a thin ossified margin, faint ground-glass density, and clump-like internal calcification (case 9).

#### Mandibular Lesions (Imaging Available for Seven Cases)

**Fig. 4:**
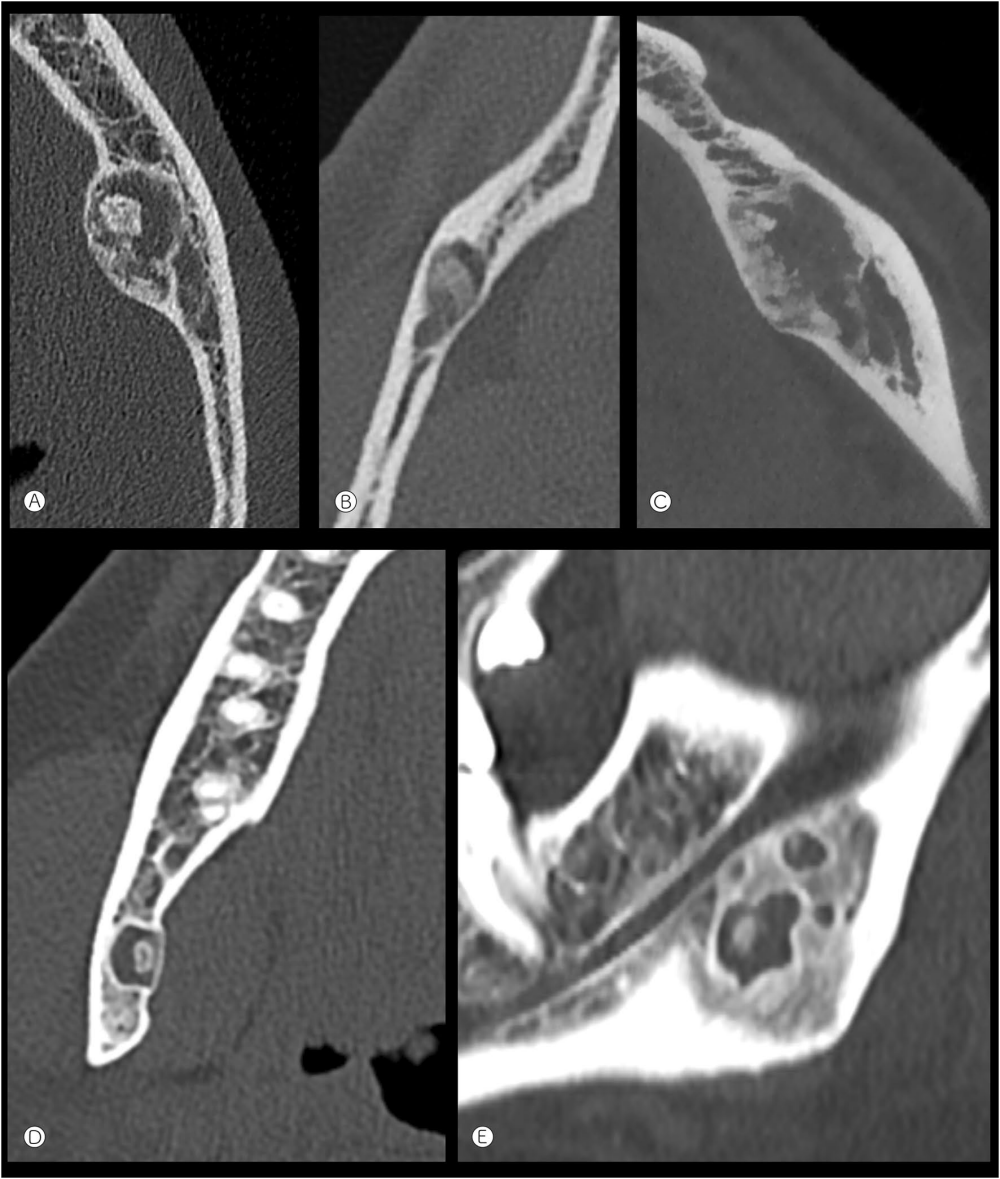
Mandibular lesions. **A** Intracortical tumor in the body of the left mandible with a thin sclerotic margin and amorphous central calcification (case 16, axial CT). **B** Intramedullary tumor in the body of the right mandible showing a thin sclerotic border, endosteal scalloping and focal ground glass density centrally (case 17, axial CT). **C** Intramedullary expansile tumor in the left mandible with a peripheral margin of sclerosis (similar pattern to several of the frontal lesions, case 15) (axial CT). **D** and **E** Axial CT (**D**) and sagittal reconstruction (**E**) showing a tumor with atypical features at the angle of the right mandible. Multilocular lucencies with thin sclerotic margins, central mineralization, cortical thinning and expansile remodeling of the medial cortex adjacent to the larger lesion. There is adjacent medullary sclerosis (case 19)

Most cases were intramedullary, two appearing intracortical (Fig. 4A). Tumors were located in the mandibular body or angle and were typically small, well-defined lucent lesions with a thin sclerotic margin, containing variable matrix mineralization: punctate calcification, ground glass density, and amorphous ossifications (Fig. 4B). None of these lesions destroyed the cortex, but there was expansile remodeling in all but two cases. Only one lesion (case 10) showed a densely mineralized margin (similar to the frontal tumors), while also demonstrating endosteal scalloping and amorphous calcification (Fig. 4C); another case (case 7) showed a mandibular angle multilocular lucency. Each locule appeared sharply marginated with a thin sclerotic border, central mineralization and cortical thinning, with adjacent medullary sclerosis (Fig. 4D, E).

#### Maxillary Lesions (Imaging Available for Two Lesions)

Both tumors were intramedullary. One occurred in the alveolar process of the maxilla and showed similarity to the frontal lesions, with thick peripheral density, central mineralization and thinning of the adjacent medial cortex (case 18). The other case was in the anterolateral wall of the maxillary antrum, showing diffuse medullary sclerosis and expanding the thin sinus wall (case 19).

#### Relationship to Dentition

**Fig. 5:**
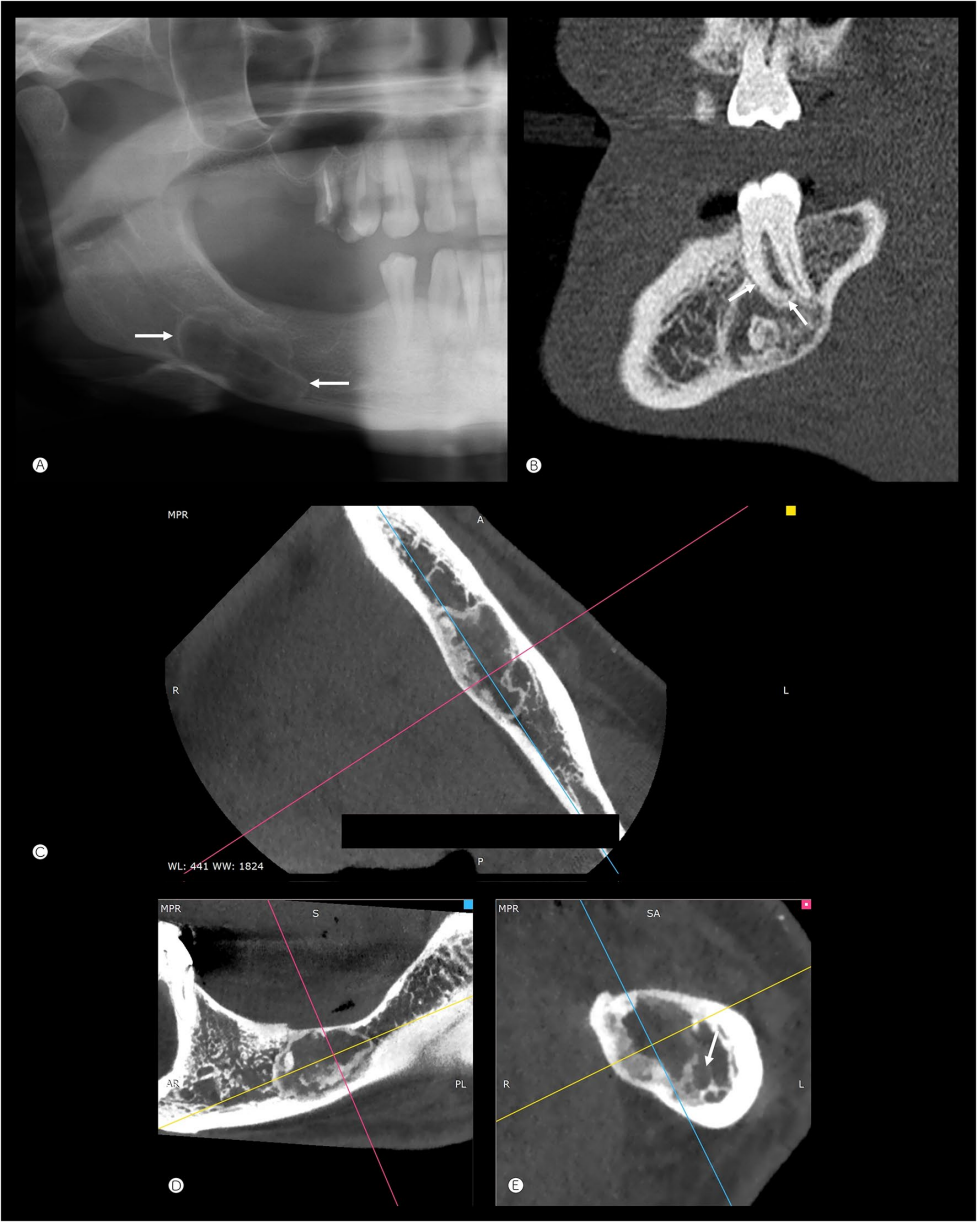
Relationship to tooth-bearing areas. **A** A lesion with a thin sclerotic margin (arrows) is predominantly inferior to the mandibular canal, scalloping the adjacent inferior cortex of the bone (case 20). **B** Sagittal CT reconstruction of the left mandible (case 16) showing remodeling of the tooth root but an intact periodontal ligament space (arrows) and lamina dura. **C**–**E** Multiplanar reconstructed CT of a lesion in the body of the left mandible (case 15) involving the edentulous tooth-bearing region. The mandibular canal is located inferolateral to the tumor (arrow in bottom right (oblique coronal) image)

Five of seven mandibular and one of two maxillary tumors involved tooth-bearing areas. For the mandibular lesions, the tumor was completely (n = 2) or predominantly (n = 1) inferior to the mandibular canal (containing the inferior alveolar nerve), suggesting a non-odontogenic origin. In two other cases, the canal passed through the mid-lateral aspect of the tumor (also suggesting a non-odontogenic origin) and in the remaining two cases, the tumor was superior or predominantly superior to the mandibular canal. In two of five mandibular tumors involving tooth-bearing areas, the periodontal ligament space appeared preserved; in the other three cases, the tumor either extended to an edentulous area of bone or a clear determination could not be made. One maxillary tumor was located in the alveolar process adjacent to teeth (Fig. 5).

### Additional and Molecular Findings

Due to tissue availability and preservation (affected by acid decalcification) only a subset of cases were suitable for additional analyses. Immunophenotyping showed faint and variable expression of smooth muscle actin in some of the cases, while desmin, S100 protein, STAT6, MDM2, and β-catenin were consistently negative (n = 14). The proliferation index (determined by Ki-67) was low in all cases analyzed (about 1% positive nuclei). *GNAS* testing did not detect any point mutation in the nine cases analysed. Five tumors were comprehensively analyzed by gene panel DNA sequencing (135 genes, including *GNAS*), Archer FusionPlex sequencing (53 genes as well as partner genes), as well as methylation and copy number profile analysis. In one case (case 12), DNA and Archer FusionPlex sequencing failed due to technical reasons, while in the remaining four cases, no fusion transcript was detected. DNA sequencing revealed four variants of unknown significance (VUS) according to the ClinVar database in three of four tumors [FANCA p.T126R, allelic frequency (AF) 50%; NOTCH1 p.R912W, AF 46%; ATM p.A1670V, AF 46%; NF1 p.G633R, AF 48%]. The methylation profiles of these cases were compared to 54 reference sets of bone and soft tissue tumors [8]. Using graphical representation (t-sne), all five cases clustered in a group distinct from the reference set of fibrous dysplasia (n = 14) but were closely related to the reference set of osteoblastoma (n = 24). Reference sets for COF, JTOF, JPOF and COD were not available for comparison. All copy number profiles were consistently flat.

## Discussion

In extragnathic sites of the craniofacial skeleton craniofacial fibrous dysplasia (CFD), juvenile psammomatoid ossifying fibroma (JPOF), and juvenile trabecular ossifying fibroma (JTOF) comprise the spectrum of benign fibro-osseous lesions according to the 2017 WHO classification since cemento-osseous dysplasia (COD) and cemento-ossifying fibroma (COF) are considered of odontogenic origin and limited to the tooth-bearing areas of the jaws [6, 7]. CFD usually shows a typical imaging presentation as it can affect contiguous bones and causes expansile remodeling. In early stages, CFD appears primarily radiolucent but becomes increasingly radiodense over time. In the peripheral skeleton, uniform ground glass opacities are usually observed, while lesions in the craniofacial bones frequently reveal more mixed lucent, dense, and occasionally cystic patterns of mineralization [9]. Patients commonly present in their first two decades of life and report painless and usually asymmetric swellings. Depending on the site of involvement, displacement of teeth and narrowing of nerve canals can cause a variety of symptoms [5]. JTOF is most common in the maxilla and only rarely involves extragnathic sites, with a mean age of presentation between 8.5 and 12 years. JPOF on the other hand, typically develops in the paranasal sinuses (70% of cases) and occurs in patients ranging from 16 to 33 years of age. It is rather rare in other bones of the skull and jaw [2]. Both JTOF and JPOF are locally aggressive and expansile tumors with a central lucency and peripheral density seen on imaging studies.

Histologically, CFD shows a mature fibroblastic stroma with immature woven bone formation, often with a peculiar curvilinear architecture. Osteoblastic rimming is typically absent and over time, lamellar maturation of the lesional trabeculae can be found. The cells carrying the *GNAS* mutation get progressively rarefied due to apoptosis, resulting in negative mutation testing in long-standing FD. JTOF is characterized by a particularly immature bone matrix that seems to directly blend into the adjacent stromal cells, which may be faint and underestimated by H&E stains. The matrix of JPOF is peculiar, characterized by small spherical ossicles rimmed by flattened osteoblasts. They are also referred to as psammomatoid bodies and may coalesce. The stromal cell component of both JTOF and JPOF appears fibroblastic and lacks pleomorphism.

The imaging appearances of the tumors in this study were variable, but usually suggested a non-aggressive fibro-osseous lesion. In the frontal bone, there was most frequently an expansile intramedullary lucency, a homogeneously dense peripheral margin, and often matrix mineralization. Occasional cortical thinning or destruction suggested a more locally aggressive tumor. Mandibular lesions in this study were also most commonly intramedullary and although the majority of cases affected tooth-bearing areas, most were either inferior to, or at the level of the mandibular canal/inferior alveolar nerve, suggesting they were of non-odontogenic origin. Five out of eight gnathic cases were not associated with teeth.

Morphologically, the cases presented here differ from the well-defined fibro-osseous lesions described. They were composed of hypercellular fibroblastic spindle cells with apparent cellular crowding. Although CFD can undergo regressive changes and show some degree of histologic diversity, the spindle cells are usually more plump and lack the slender and tapered nuclei found in this series of cases. The lesional matrix consisted of abundant small fragments of particularly hypocellular woven bone and lacked the trabecular architecture seen in CFD. Mutation testing showed absence of GNAS mutations in nine analysed cases, further arguing against a molecular relation with CFD. JTOF and JPOF can show a similar spindle cell component although again the slim and sometimes wavy appearing nuclei are not a typical finding. The lesional matrix defines JTOF and JPOF morphologically and is distinct from the hard tissue formation in the lesions described herein. Particularly JPOF, which show similarities in anatomic-topographic distribution, present with abundant psammomatoid bodies resembling dental cementum and usually results in a rather monomorphic appearance. JPOF can occur in adults but patients are nevertheless significantly younger than those described here (mean 44 years). COF typically shows rimming of activated osteoblasts and occasionally a matrix-free rim at the periphery separating the lesion from preexisting bone, neither of which was observed in the current cases [3, 12]. COD typically is centered around the teeth roots and in the majority of cases lacks an expansile growth, a finding distinctly different from the presented cases. Central low-grade osteosarcoma, exceptionally rare in the head and neck, can appear histologically similar to CFD but is usually characterized by a more aggressive imaging appearance and cellular atypia.

Taken together, the presented cases seem distinct from the classical subtypes of craniofacial fibro-osseous lesions described in the current WHO classification of head and neck tumors. Since no recurrent genetic aberration has been identified in any of the fibro-osseous lesions with the exception of CFD, the classification is based mainly on anatomic-topographic distribution, imaging, patient characteristics and microscopical appearance. The extragnathic cases are clearly of non-odontogenic origin and we provide evidence that also the presented tumors from the mandible and maxilla cannot be unequivocally categorized as COF or COD despite some degree of morphologic overlap. As long as no additional molecular data suggests the cases from our series to belong to a separate fibro-osseous subtype, we propose to broaden the criteria defining ossifying fibroma and to consider revising the name from cemento-ossifying fibroma to conventional ossifying fibroma comprising odontogenic and non-odontogenic subtypes. However, this opinion is not shared by other authors who recently proposed to more strictly separate COF as an odontogenic neoplasm from the juvenile, non-odontogenic subtypes of OF [3]. Alternatively, a new category of ossifying fibroma of non-odontogenic origin might be required.

## Supplementary Information

Below is the link to the electronic supplementary material.

Supplementary file1 (DOCX 19 kb)

Supplementary file2 (TIF 4586 kb)

Supplementary file3 (TIF 5099 kb)
