# Supplementary material for: Ossifying Fibroma of Non-odontogenic Origin: A Fibro-osseous Lesion in the Craniofacial Skeleton to be (Re-)considered
Source: Head Neck Pathol. 2021 Jun 26;16(1):257–67. doi: 10.1007/s12105-021-01351-3 (PMC9018933; doi:10.1007/s12105-021-01351-3)
Supplement: Supplementary file 1 — Supplementary file1 (DOCX 19 kb) [file 12105_2021_1351_MOESM1_ESM.docx]

**Supplementary data**

*Tissue selection, DNA extraction and sequencing*

For DNA and RNA isolation, the area of interest was marked on H&E stained tissue slides and then macrodissected by scratching. The DNA was isolated using the Maxwell DNA purification kit according to the manufacturer’s protocol. The DNA concentration was quantified using the Qubit® dsDNA HS Assay Kit (ThermoFisher Scientific). The Ion AmpliSeq Library Kit V.2.0 (ThermoFisher Scientific) was used to prepare the libraries from 10 ng of DNA. The Ion Ampliseq HiFi Master Mix (ThermoFisher Scientific) was used to prepare the amplicons that were digested with FUPA reagent and tagged with the IonCode Barcode Adapters. Finally, the amplified products were purified using the Agencourt AMPure XP PCR purification system (Beckman Coulter, California, USA). The Ion Library Equalizer Kit method was used to normalize the library concentration at ~100 pM. Finally, equal volumes of normalized DNA library were combined and amplified on Ion Sphere particles (ISP; ThermoFisher Scientific) by emulsion PCR using the Ion PI HiQ OT2 200 Kit (both ThermoFisher Scientific). Quality control was performed using the Ion Sphere Quality Control kit (ThermoFisher Scientific) to ensure that 10%–30% of template positive ISP were generated in the emulsion PCR. The template-positive Ion PI ISP were loaded on an Ion PI Chip and sequenced on an Ion S5^TM^ XLSequencer (ThermoFisher Scientific) with the Ion PI HiQ Sequencing 200 Kit (ThermoFisher Scientific) according to the manufacturer’s instructions. For NGS data analysis, the Ion Reporter Software within Torrent Suite Software (ThermoFisher Scientific) was used. Detected sequence variants were evaluated for their pathogenicity based on previous literature, databases (COSMIC, ClinVar). Mutations classified as benign were not reported.

*Immunohistochemistry*

Immunohistochemical analyses were carried out according to standard protocols and included the following antibodies: Ki-67 (clone MIB-1, IR626, Dako, Glostrup, Denmark), MDM2 (clone IF2, AB_2533136, Thermo Fisher Scientific, Allschwil, Switzerland), STAT6 (clone YE361, ab32520, abcam, Cambridge, UK), SM-Actin (clone 1A4, 760-2833, Roche Diagnostics, Rotkreuz, Switzerland), Desmin (clone DE-R-11, 760-2513, Roche Diagnostics, Rotkreuz, Switzerland), S100 protein (clone 4C4.9, 790-2914, Roche Diagnostics, Rotkreuz, Switzerland), and ß-catenin (clone 14, 760-4242, Roche Diagnostics, Rotkreuz, Switzerland).

*RNA isolation and sequencing*

Total RNA was extracted by using the Maxwell® RSC RNA FFPE Kit (Promega, Madison, USA). Libraries were prepared using a customized RNA-based NGS panel (Archer^TM^ FusionPlex^TM^, ArcherDx, Inc., Boulder, CO), containing 63 genes known to be translocated in tumors. 250ng of RNA was used for generating NGS libraries, which were loaded to an Ion 540^TM^ chip (Thermo Fisher Scientific) for sequencing. Raw data were processed automatically on the Torrent Server^TM^ v5.10 and aligned to the hg19 reference genome. Bam files were uploaded into the Archer data analysis pipeline (Archer^TM^ analysis software version 6.0).

*Genes included in the Oncomine^TM^ Comprehensive Panel*

*Hotspots:* AKT1, AKT2, AKT3, ALK, AR, ARAF, AXL, BRAF, BTK, CBL, CCND1, CDK4, CDK6, CHEK2, CSF1R, CTNNB1, DDR2, EGFR, ERBB2, ERBB3, ERBB4, ERCC2, ESR1, EZH2, FGFR1, FGFR2, FGFR3, FGFR4, FLT3, FOXL2, GATA2, GNA11, GNAQ, GNAS, H3F3A, HIST1H3B, HNF1A, HRAS, IDH1, IDH2, JAK1, JAK2, JAK3, KDR, KIT ,KNSTRN, KRAS, MAGOH, MAP2K1, MAP2K2, MAP2K4, MAPK1, MAX, MDM4, MED12, MET, MTOR, MYC, MYCN, MYD88, NFE2L2, NRAS, NTRK1, NTRK2, NTRK3, PDGFRA, PDGFRB, PIK3CA, PIK3CB, PPP2R1A, PTPN11, RAC1, RAF1, RET, RHEB, RHOA, ROS1, SF3B1, SMAD4, SMO, SPOP, SRC, STAT3, TERT, TOP1, U2AF1, XPO1.

*Full genes:* ARID1A, ATM, ATR, ATRX, BAP1, BRCA1, BRCA2, CDK12, CDKN1B, CDKN2A, CDKN2B, CHEK1, CREBBP, FANCA, FANCD2, FANCI, FBXW7, MLH1, MRE11A, MSH2, MSH6, NBN, NF1, NF2, NOTCH1, NOTCH2, NOTCH3, PALB2, PIK3R1, PMS2, POLE, PTCH1, PTEN, RAD50, RAD51, RAD51B, RAD51C, RAD51D, RB1, RNF43, SETD2, SLX4, SMARCA4, SMARCB1, STK11, TP53, TSC1, TSC2.

*Genes included in the Oncomine^TM^ Colon Panel*

*Hotspots:* AKT1, APC, BRAF, CTNNB1, EGFR, ERBB2, FBXW7, GNAS, KRAS, MAP2K1, NRAS, PIK3CA, SMAD4, TP53

*Genes included in the Archer^TM^ FusionPlex^TM^ Custom Panel*

ACVR2A, ALK, BCOR, BRAF, CAMTA1, CCNB3, CIC, CSF1, EGFR, EPC1, ERG, ETV1, EWSR1, FGF1, FGFR1, FGFR2, FGFR3, FGR, FOS, FOSB, FOXO1, FUS, GLI1, GRM1, HMGA2, IGF1R, JAZF1, MAML2, MEAF6, MET, MGEA5, MKL2, MSANTD3, MYBL1, MYB, NCOA2, NFATC2, NOTCH1, NRG1, NTRK1, NTRK2, NTRK3, NUTM1, PAX5, PDGFB, PHF1, PIK3CA, PLAG1, PPARG, PRKD1, RAF1, RET, ROS1, SRF, SS18, STAT6, TAF15, TCF12, TFE3, TFG, TMPRSS2, USP6, YWHAE

*Methylation and copy number profiling*

Approximately 1μg of DNA, determined by absorption (Nanodrop method) and representing a tumor area of approximately 0.5 mm^2^, was used for the analysis. The DNA methylation profile was then obtained on an Infinium Human Methylation EPIC BeadChip array (Illumina, USA). The resulting IDAT files were processed using the in-house diagnostic toolchain EpiDiP (publicly available at [http://www.epidip.org](http://www.epidip.org/)) that allows comparison of a given dataset against currently more than 15,500 reference datasets mostly derived from The Cancer Genome Atlas (TCGA) and Gene Expression Omnibus (GEO). In addition, raw data of diagnostically worked up cases from within our institution and collaborators have been injected into this data lake. The analysis considers both the DNA methylation profile and the copy number variations. This software is primarily written in R (version 3.6.2 as of the time of writing this manuscript) and solely relies on open-source packages. IDAT data, the format provided by Illumina Methylation Array scanners, are parsed through minfi (available from Bioconductor) and normalized using the SWAN algorithm (Bioconductor). Filtering for the top differentially methylated probes is performed through a standard deviation of all probes across all datasets. Only the top 25,000 probes of this ranking, adopted from the data preparation method used to build the brain tumor methylation classifier, are considered for dimension reduction by uniform manifold approximation and projection (UMAP, R implementation, available from CRAN). Specimens sharing epigenomic and hence lineage similarities cluster together as demonstrated previously with t-distributed stochastic neighbor embedding (t-SNE). Genome-wide copy number alterations are read out through conumee (available on Bioconductor). Copy number plots are calculated for all cases in the reference data lake and made accessible for each case through EpiDiP. The pan-data-lake UMAP plot is overlaid with tissue type annotations curated in an in-house database and linked to copy number plots for each sample. These data are presented to the pathologist through a Shiny-based web application that we made available for free online ([http://www.epidip.org](http://www.epidip.org/)).
