# Supplementary figures and images for: Ossifying Fibroma of Non-odontogenic Origin: A Fibro-osseous Lesion in the Craniofacial Skeleton to be (Re-)considered
Source: Head Neck Pathol. 2021 Jun 26;16(1):257–67. doi: 10.1007/s12105-021-01351-3 (PMC9018933; doi:10.1007/s12105-021-01351-3)

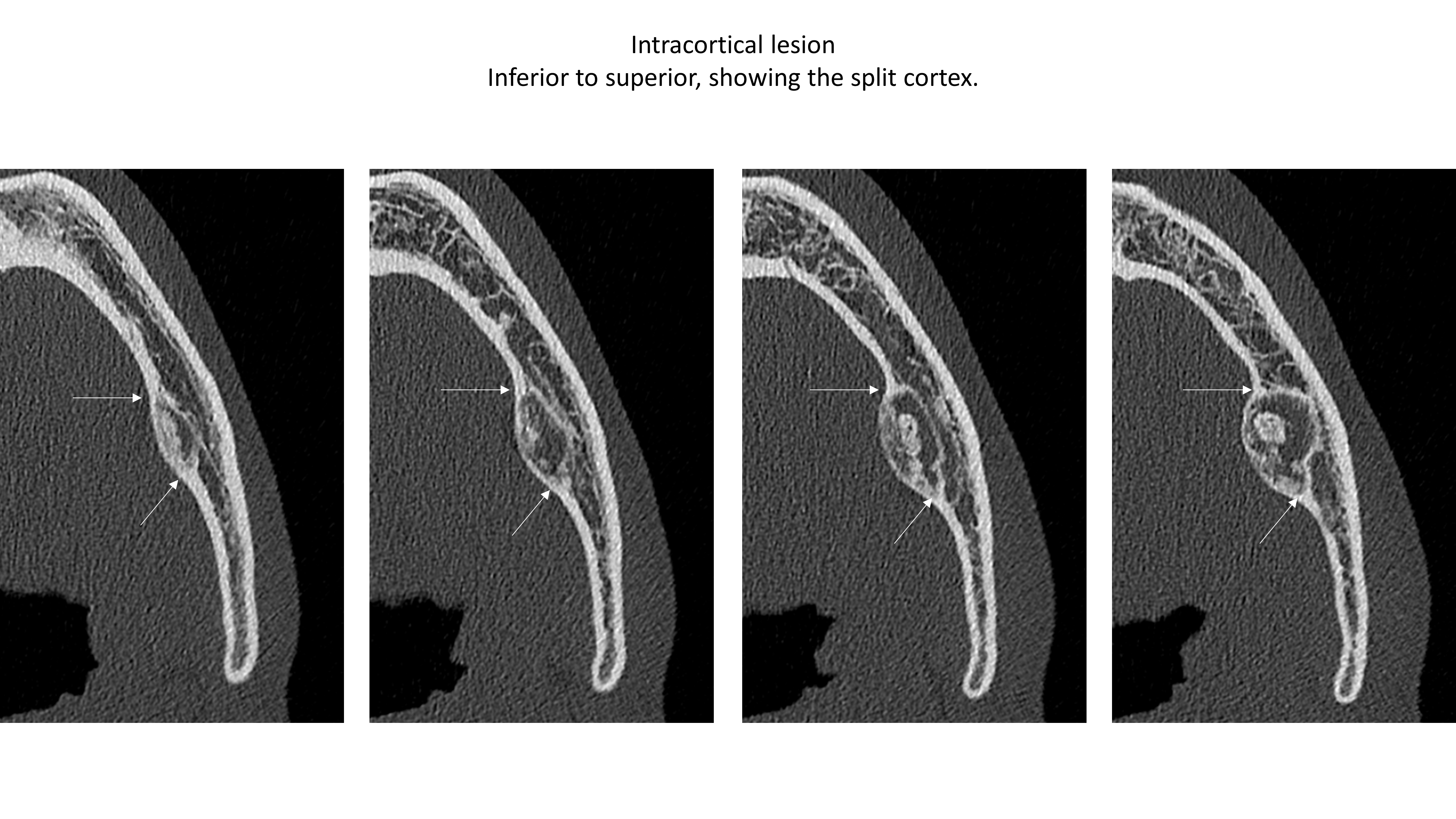

Supplement: Supplementary file 2 — Supplementary file2 (TIF 4586 kb) [file 12105_2021_1351_MOESM2_ESM.tif]

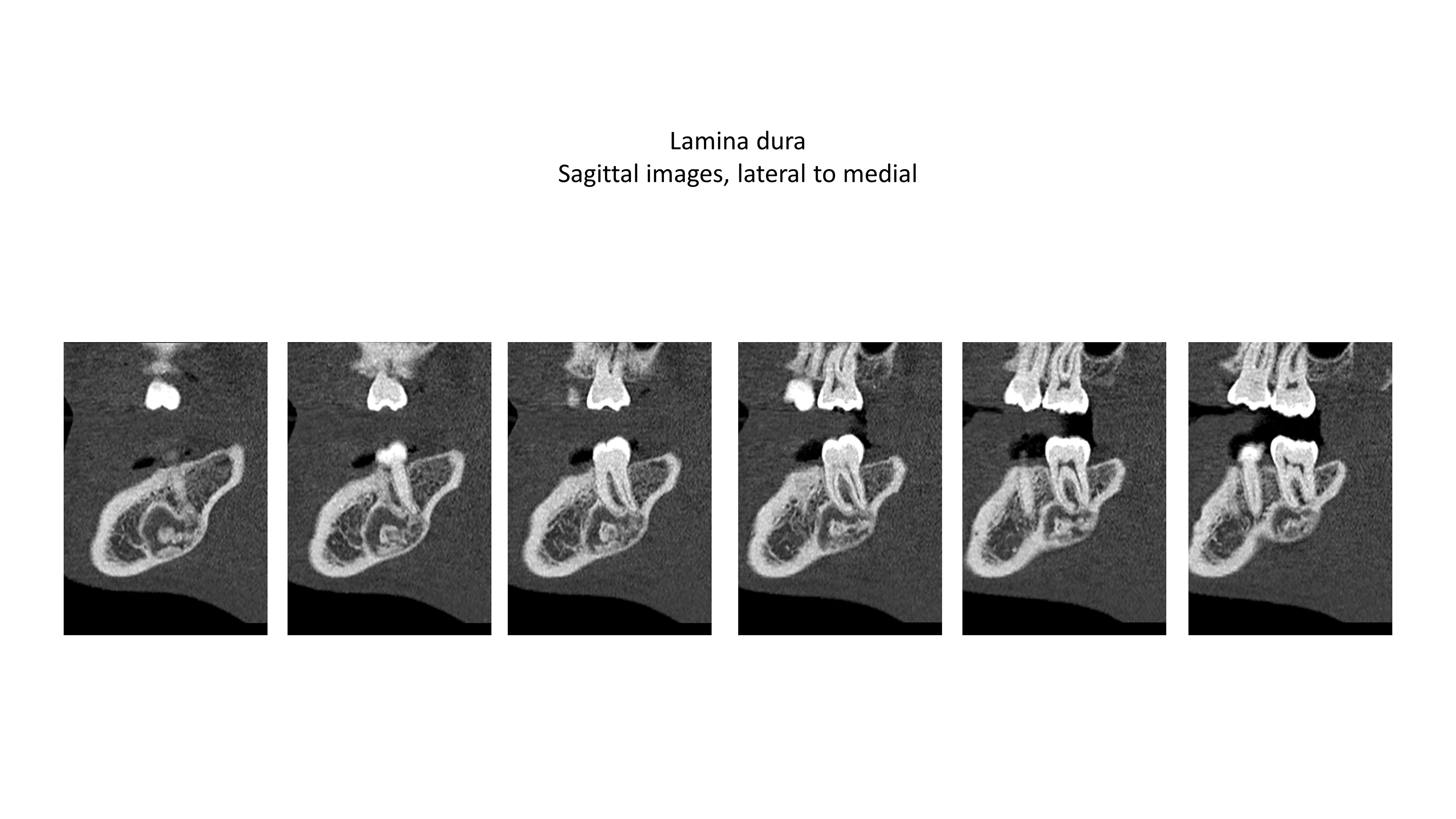

Supplement: Supplementary file 3 — Supplementary file3 (TIF 5099 kb) [file 12105_2021_1351_MOESM3_ESM.tif]
